# Letter to the Editor in Response to ‘Population-based cohort study of oral contraceptive use and risk of depression’

**DOI:** 10.1017/S2045796024000039

**Published:** 2024-02-01

**Authors:** P. Kendall, A. Lazorwitz

**Affiliations:** 1 Department of Obstetrics and Gynecology, Division of Family Planning, University of Colorado School of Medicine, Aurora, CO, USA; 2 Women’s Reproductive Health Research Scholar, Department of Obstetrics and Gynecology, Division of Family Planning, University of Colorado Anschutz Medical Campus, Aurora, CO, USA

Dear Editor-in-Chief,

We read with interest the article by Johansson *et al.* published first online on 12 June 2023 in *Epidemiology and Psychiatric Sciences* (Johansson *et al.*, 2023). In this population-based cohort study, the authors evaluated for a causal relationship between depression and oral contraceptive (OC) use. While the authors included data from over 250,000 participants from the UK Biobank with extensive data points, the authors failed to adequately address two major limitations of their study: (1) reported OC use primarily from the 1970s and 1980s with outdated OC preparations and (2) no acknowledgment of a potential ‘nocebo’ effect.

OCs have changed dramatically over the decades since the first pill became available in 1961 (Dhont, 2010). As the authors mentioned, the majority of combined oral contraceptives (COCs) available in the United Kingdom during the majority of reported OC use in this study included 100–150 μg of the second-generation progestin, levonorgestrel, in combination with 20, 30 or 50 μg of ethinylestradiol. Levonorgestrel, a derivative of 19-nortestosterone, has one of the highest affinities for the progesterone receptor among progestins, but likewise also one of the highest affinities for the androgen receptor, leading to increased androgenic side effects (Dhont, 2010). Subsequently developed third- and fourth-generation progestins have anti-androgenic properties while maintaining beneficial properties such as potency and longer half-lives, making COC formulations containing third- and fourth-generation progestins better options for the vast majority of patients today (Dhont, 2010). While the exact relationship between OC and mood is poorly understood, the results of several studies have suggested that the androgenicity of the progestin in a COC may be the most influential factor (Schaffir *et al.*, 2016). Randomized trials have demonstrated higher rates of adverse mood symptoms among patients taking a pill with levonorgestrel compared to patients taking pills containing either desogestrel, a third-generation progestin, or drospirenone, a fourth-generation progestin (Kelly *et al.*, 2010; Sangthawan and Taneepanichskul, 2005; Schaffir *et al.*, 2016; Shahnazi *et al.*, 2014). Further, the authors do not distinguish between the use of progestin-only OCs or COCs in their analyses. Progestin-only OCs were available during the time frame of this cohort study, and yet the authors provide no justification for lumping non-oestrogen containing OC formulations with oestrogen-containing formulations, despite the hypothesized effect of oestrogens on mood symptoms.

Finally, as first introduced by Grimes and Schulz in 2011, we know that placebo-controlled randomized trials have actually demonstrated that many mood-related symptoms reported by OC users are due to a ‘nocebo’ effect (Grimes and Schulz, 2011). As patients prescribed OCs are counselled to expect adverse mood symptoms, they then experience higher rates of these adverse side effects (Grimes and Schulz, 2011). As such, the appropriate comparison group for evaluating side effects, including mood disorders, due to OC use is a true placebo group (users of inert pills) and not a never OC user group as utilized by the authors.

We fully support the evaluation of safety and side-effects with hormonal contraceptive options as these studies can improve patient counselling. Unfortunately, research that demonstrates increased risks of side effects with hormonal contraceptive use, especially mood disorders, must be scientifically rigorous to avoid unnecessary public backlash and patient avoidance of reliable contraceptive options. We applaud the efforts by Johansson *et al.* to tackle this difficult area of research but must express caution in generalizing the findings from this study to more modern OC formulations, and these findings must be put into the context of the known ‘nocebo’ effect with hormonal contraception.

Sincerely,

Paige Kendall, MD

Aaron Lazorwitz, MD, PhD, MSCS
